# Inkjet printed self-healable strain sensor based on graphene and magnetic iron oxide nano-composite on engineered polyurethane substrate

**DOI:** 10.1038/s41598-020-75175-6

**Published:** 2020-10-26

**Authors:** Gul Hassan, Muhammad Umair Khan, Jinho Bae, Ahmed Shuja

**Affiliations:** 1 Department of Ocean System Engineering, Jeju National University, 102 Jejudaehakro, Jeju, 63243 South Korea; 2 Centre for Advanced Electronics and Photovoltaic Engineering (CAEPE), International Islamic University, H-10, Islamabad, 44000 Pakistan

**Keywords:** Chemical engineering, Electrical and electronic engineering, Materials for devices

## Abstract

In recent years, self-healing property has getting tremendous attention in the future wearable electronic. This paper proposes a novel cut-able and highly stretchable strain sensor utilizing a self-healing function from magnetic force of magnetic iron oxide and graphene nano-composite on an engineered self-healable polyurethane substrate through commercialized inkjet printer DMP-3000. Inducing the magnetic property, magnetic iron oxide is applied to connect between graphene flacks in the nano-composite. To find the best nano-composite, the optimum graphene and magnetic iron oxide blending ratio is 1:1. The proposed sensor shows a high mechanical fracture recovery, sensitivity towards strain, and excellent self-healing property. The proposed devices maintain their performance over 10,000 times bending/relaxing cycles, and 94% of their function are recovered even after cutting them. The device also demonstrates stretchability up to 54.5% and a stretching factor is decreased down to 32.5% after cutting them. The gauge factor of the device is 271.4 at 35%, which means its sensitivity is good. Hence, these results may open a new opportunity towards the design and fabrication of future self-healing wearable strain sensors and their applied electronic devices.

## Introduction

In recent days, flexible strain sensors have got tremendous attention in the various applications such as human motion detection^1^, health care^2^, damage detection^3^, characterization of structures^4^, and exhaustion studies of materials^5^. For these kinds of applications, well established strain sensors are required. However, the micro cracks and mechanical fractures in the strain sensors are very repeatedly occurred under repeated deformation^6^. The performance of a strain sensor is susceptible to the structure damage, and this damage may cause the loss of a functionality of the sensor^7^. Therefore, it is necessary to introduce a sensor with self-healing property to improve its reliability, and to maintain its function towards the strain through its healing even though it is cut.

To overcome the fracture problem of the strain sensors, many research groups are exploring and developing self-healing conductors and sensors by using the various materials and nano-composites^8,9^. The self-healing materials are smart materials that can restore some or all of its functions after cutting or suffering by an external damage from mechanical force^10^. Their self-healing property are not only prolonging the life time of products but also reducing the wastage of the materials^11–13^. Especially, this function increases their reliability of the strain sensor based devices^14^. To cover these good advantages, self-healing mechanism and several materials have been studied^15–22^. In terms of materials, two types of materials have been reported in early decades with intrinsic self-healing and extrinsic self-healing properties^11,18,23–26^. Self-healing property in intrinsic materials is due to the reversible covalent and non-covalent bonds^27–33^ while self-healing in extrinsic materials is due to microcapsules or healing agent, which heals the cracks when cracks are formed due to external strain^34–40^. The self-healing property of these materials is not just limited to a mechanical performance. It is utilized for the various application such as conductivity^41,42^, artificial electronic skin^43^, medical devices^44^, and soft robotics^45–47^. For the strain sensor applications, the self-healing property as well as a good electric conductivity should be satisfied^24,44,48–50^.

Herein, this paper proposes a novel high stretchable strain sensor with the self-healing property depositing graphene and magnetic iron oxide nano-composite on the engineered self-healable polyurethane substrate through Fujifilm inkjet DMP-3000 printer at ambient conditions as shown in Fig. S1 of supplementary information. To include a self-healing function between graphene flake to flake, magnetic iron oxide is blended, and it has also a role to induce the magnetic force property in the nano-composite. Experimentally, the best graphene and magnetic iron oxide blending ratio is 1:1. This composite material shows a high mechanical and excellent self-healing property. To increase a self-healing property of the proposed sensor, the engineered self-healable polyurethane substrate is applied due to its strong self-healing function. The prepared samples maintain its strain function of 100% even after 10,000 times bending cycles, and the strain sensitivity performance of 94% recovered after cutting the sensor. The proposed strain sensor is stretchable up to 54.5%. After cutting it, the stretching factor decrease down to 32.5%, but it still working good for a strain sensor application. For these results, we are sure that this work can create a new opportunity towards the design and fabrication of a future self-healing wearable strain sensing based electronic devices.

## Materials and methods

### Materials preparation

Graphene flacks with average size of 100 nm, heptane solvent, magnetic iron oxide (Fe_2_O_3_) nano-particles solution in heptane with 0.8–1.4% solid material was purchased from Sigma Aldrich, South Korea. To make the ink solution based on graphene, 0.5 mg graphene was dispersed in 4 ml heptane solvent. The ink was sonicated for 30 min at room temperature to make homogenous suspension. The solution was then centrifuged for 30 min at 1000 rpm to remove the big chunks and particles. The super latent was removed in a vile, which was used as the final solution for the fabrication of active layers. Magnetic iron oxide solution was used without further purification. For the best fabrication of the composite film, both inks were mixed with different blending ratios. To find the best blending ratio, different ratios of graphene and Fe_2_O_3_ were used, i.e. 1:0, 1:0.5, 1:1, and 1:1.5. The solution was bath sonicated for 30 min at room temperature to make homogenous composite solution and then put on shaker for 1 h. The viscosity of the composite ink was measured to be 12.35–16.9 mPa by using Viscometer VM-10A system. The surface tension was measured 41–54 mN/m by Surface electro-optics (SEO)’s contact angle analyzer.

### Polyurethane substrate preparation

The wrinkled aluminium foil substrate was prepared by squeezing and unfolding the 60 mm × 60 mm × 10 µm aluminium foils. This methodology can create random micro ridges on the aluminium foil, which is finally suppressed by using a soft hammer^51^ as shown in Fig. S1a of supplementary information. After that, the wrinkled aluminium foil was attached to the glass substrate using a double-sided stick tape to caste a liquid polyurethane. The casted polyurethane was cured inside UV ozone for 5 min. The cured polyurethane was cut into different substrate sizes to use for the sensor. The prepared polyurethane substrate with the random micro ridges has several advantages as it ensures the stickiness of the active film and also helps in scattering the applied strain^51^.

### Fabrication

The commercialized materials ink-jet printer (Fujifilm Dimatix DMP-3000) was utilized to fabricate the graphene and Fe_2_O_3_ nano-composite active film on the engineered polyurethane substrate. To begin with, schematic of the active layer (graphene and Fe_2_O_3_ nano-composite) was designed in EAGLE 7.4.0 and converted to bitmap image format using ACE 3000 and then exported to Dimatix Drop Manager, which converts the Bitmap image file into ptn format^51^. The resulted file was loaded into the material ink-jet printer (DMP-3000) and the graphene/Fe_2_O_3_ nano-composite ink was loaded in the cartridge containing 16 nozzles. The composite ink was deposited by using 10 pL drop size on the engineered polyurethane substrate, and the stable wave shapes for the print head was determined at 26 V, which was applied to fabricate the proposed active layer and then cured at 100 °C for two hours.. The schematic diagram of the ink-jet material printer and the real image of DMP-3000 material printer shown in Fig. S1b of supplementary information. In supplementary information, contact pads were deposited for external circuitry interfacing and all fabrication steps of the proposed strain sensor are shown in Fig. S1.

### Characterizations

**Figure 1:**
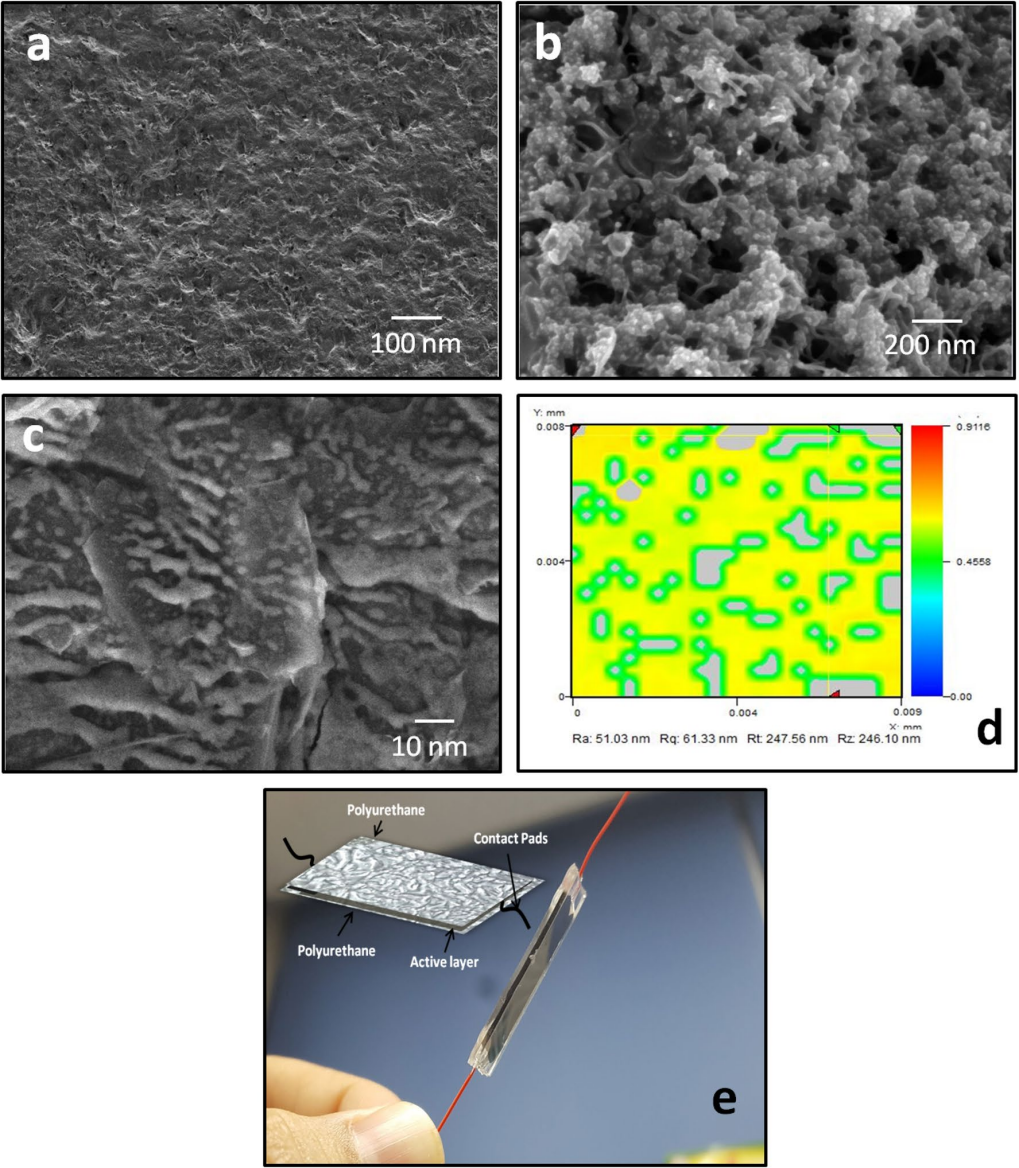
SEM Analysis of the active layer. **(a)** SEM image of graphene film, **(b)** SEM image of magnetic iron oxide film, and **(c)** SEM image of self-healing nano-composite film of graphene and magnetic iron oxide. **(d) **3D morphology of the active layer on engineered polyurethane substrate. **(e) ** The fabricated strain sensor based on graphene and magnetic iron oxide nano-composite.

Field Emission Scanning Electron Microscope (TESCAN) was utilized to examine the surface morphology of graphene, Fe_2_O_3_, and the active layer based on graphene and Fe_2_O_3_ nano-composite as shown in Fig. 1. To investigate the composite active layer further, EDS mapping analysis was performed by using Field Emission Scanning Electron Microscope (TESCAN) as shown in Fig. S2 of supplementary information. NV-2000(Universal) non-contact surface profiler with nano level accuracy was applied for roughness measurement and Fig. 1d shows the 3D profile of the active layer. The nano-composite active film is spatially uniform due to the polyurethane substrate roughness as we can see in the 3D morphology image. Electrical characterizations of the sensor was performed by using Agilent B1500A Semiconductor Device and the stretching characterization were performed by homemade stretching machine while the bending tests were also performed to verify the flexible nature of the fabricated strain sensor.

## Results and discussion

To begin with, Field Emission Scanning Electron Microscope (TESCAN) was used to analyze the surface morphology of graphene, Fe_2_O_3_, and their nano-composite active film. Figure 1a shows the SEM image of graphene and confirms that the film is uniformly deposited without visible defects. Figure 1b shows the SEM image of Fe_2_O_3_ thin film and the film is uniformly deposited, while Fig. 1c shows the SEM image of the graphene and Fe_2_O_3_ nano-composite active film as certain crack free film, which is properly deposited over engineered polyurethane substrate through ink-jet material printer (DMP-3000). As Fig. 1c is the zoomed image of the active composite film, we can clearly see that both the graphene and Fe_2_O_3_ are present in the film. In order to ensure the presence of both graphene and Fe_2_O_3_, the details are explained in Fig. S2 of supplementary information. From this analysis, we confirm the uniform and equal distribution of all materials over the entire surface of the engineered polyurethane substrate. Based on graphene and magnetic iron oxide, the proposed strain sensor is fabricated on the engineered polyurethane substrate as shown in Fig. 1e.

To enhance the stretchability of the proposed strain sensor, the substrate roughness plays a key role in stretchable electronic devices^51^ as shown in Fig. 1d, and its shows the 3D morphology of the active layer on engineered polyurethane substrate. Both the tensile stress and cracks on the film surface depend on the nature of substrate^52^. The strain endurance of the proposed thin film is enhanced by two design concepts; the substrate roughness and the addition of Fe_2_O_3_. The polyurethane substrate surface pattern was manufactured by an aluminium foil, which was chosen due to the roughness feature of the surface. Especially, it increases the adhesion between the active film layer and the substrate. We concluded that 0.34 is the best roughness to be used for strain sensor as reported in our previous work^51^. Fe_2_O_3_ has the magnetic force property, so its addition to graphene increases the stretching parameter by making the connection between the graphene flake to flake.

**Figure 2:**
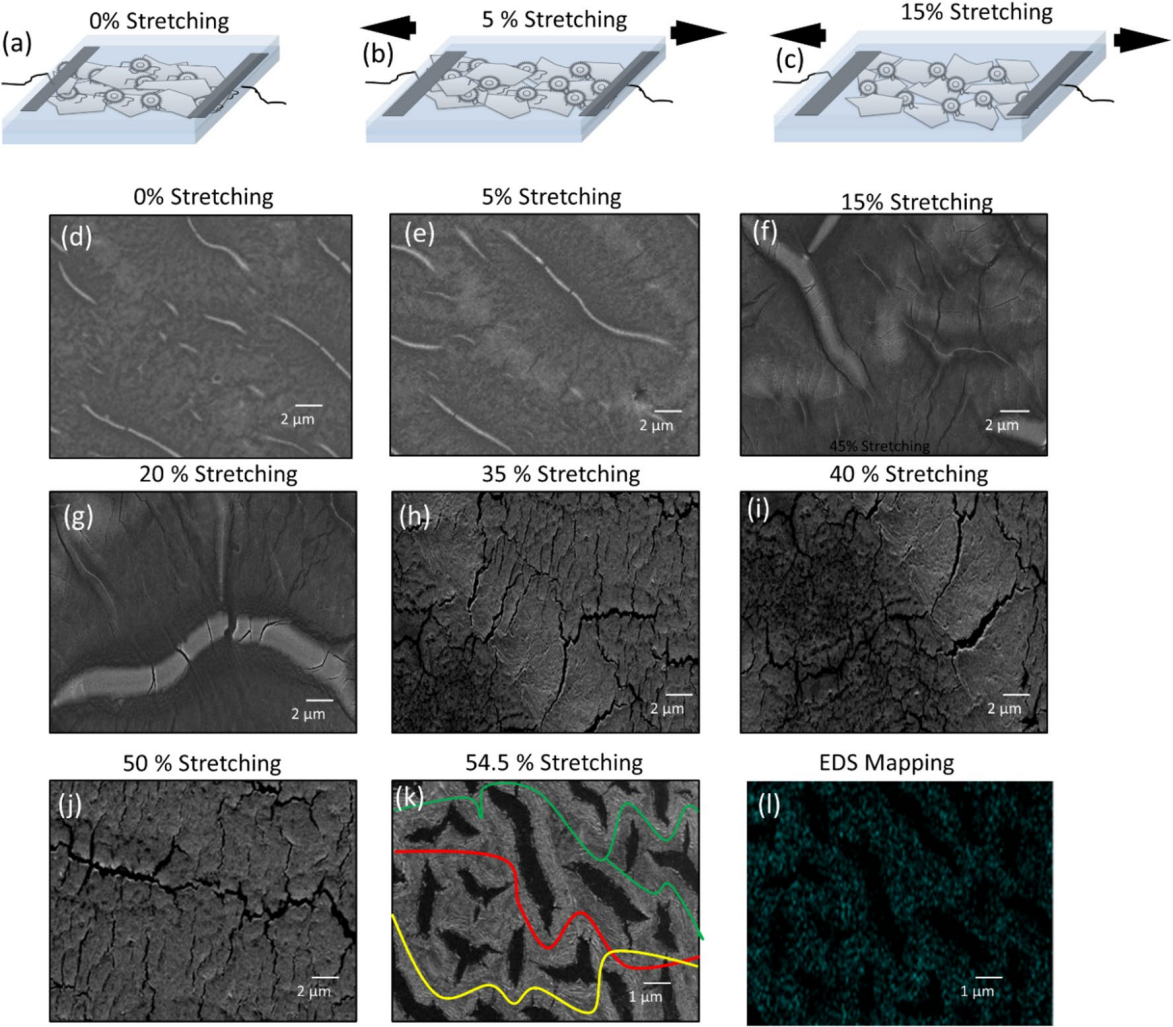
Strain detection mechanism of the proposed self-healing strain sensor. **(a)** Schematic illustration of the strain sensing with 0%. **(b) **Schematic illustration of the strain sensing with 5%, with increasing the stretching strain, the overlapping area between graphene flakes decrease that results in the increase in resistance. **(c)** Schematic illustration of the strain sensing with 15%, along the decreasing overlapping area between graphene flakes, small micro cracks also appear that results in the increase in the resistance of the strain sensor. **(d)** SEM image of the active composite film with 0% stretching. **(e) **SEM image with 5% stretching. **(f)** SEM image with 15% stretching. **(g)** SEM image with 20% stretching. **(h) **SEM image with 35% stretching. **(i)** SEM image with 40% stretching. **(j) **SEM image with 50% stretching. **(k)** SEM image with 54.5% stretching. **(l) **EDS elemental mapping after 54.5% stretching.

To demonstrate the stretching effects of the proposed strain sensor, the schematic illustrations are demonstrated as shown in Fig. 2a–c. By applying an external tensile stress, the overlapping region between flakes decreases due to graphene flakes spaced out, and the active film resistance is increased as a function of an external tensile strain. For the external tensile strain < 5% (% increase in device length), the substrate roughness acts as the wrinkled pattern and the deformation on active film remains lower than the strain on the substrate. The deformation on graphene/Fe_2_O_3_ nano-composite film remains lower than the strain force < 5% on the substrate. When the tensile strain is less than 5%, the active layer resistance increases, due to the decreasing overlapping between flakes. When the tensile strain is increased more than 5%, several small cracks appear on the nano-composite film of graphene/Fe_2_O_3_, which result in increase in resistance is observed due to moment of flakes as well as appearance of small cracks as shown in Fig. 2d–l. Here, if the stress is more increased on active layer, these cracks are nucleate due to the rough polyurethane substrate and as well presence of magnetic Fe_2_O_3_, and that prevents the breakdown of the active film due to its self-healing property. Since the roughness induces an inhomogeneous stress field on it, the destructive force is scattered as evidence shown in Fig. 2d–l. Hence, the stretchability of the active film on the micro-random ridge polyurethane substrates can be dramatically increased. Here, the stretching factor is increased due to substrate roughness and the blending of magnetic iron oxide. As magnetic iron oxide has the magnetic property, so it makes paths from flake to flake and prevent the film form breakdown. The magnetic material also performed the self-healing function on the active layer.

The resistance of the active film is increasing if the cracks size or their numbers are increasing^53^. The electrical resistance changes in two phenomena as; (1) the resistance change is dominant due to flakes movement under 5% tensile stress, (2) for more than 5% tensile stress, the resistance changes due to the formation of cracks. Although the cracks are occurred on the active film due to increase in tensile stress, it can still operate as a strain sensor because of progressive paths transformation from initial tracks into one or several longer and narrower conductive paths, which results in increasing the film resistance as shown in Fig. 2k. By increasing roughness on the polyurethane substrate, the stretchability is improved as it leads to a higher density of pinning centers which help the scattering of tensile stress all over the active film. The substrate roughness was optimized in our previous work^51^, and the obtained optimum roughness was applied.

To find the best blending ratio of graphene and magnetic iron oxide for the proposed strain sensor, we have fabricated four type of devices with different blending ratios of 1:0, 1:0.5, 1:1, and 1:1.5, and calculated the sensitivity of each ratio at different bending diameter. Under different bending diameters, the sensitivity of the devices with the same surface roughness of 0.34 was observed^51^. The following equation was used to find out the sensitivity of the devices.

1 $${S}_{R}= \frac{\Delta R}{{R}_{o}} \times 100$$

**Figure 3:**
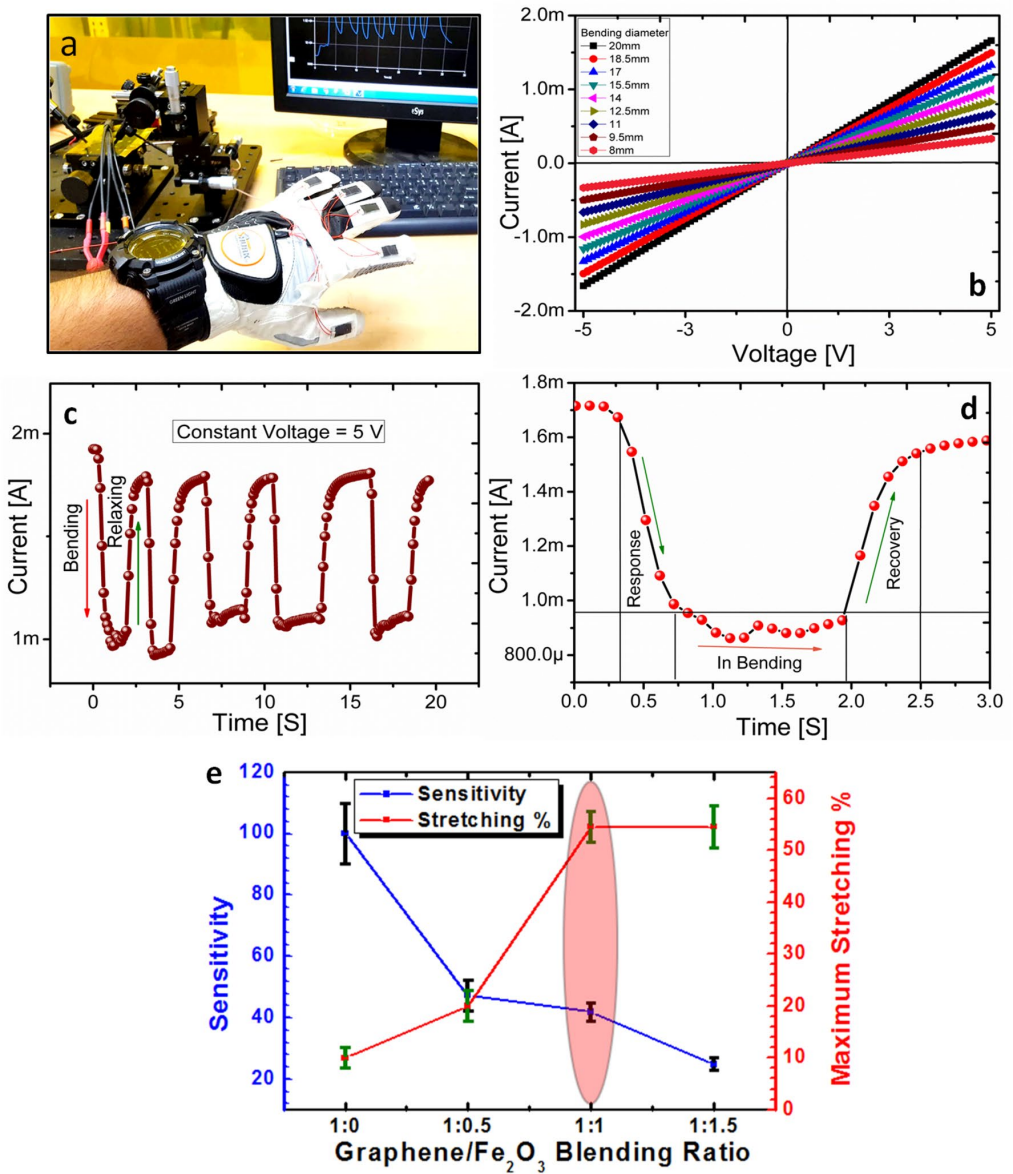
Characterization of a strain sensor based on just graphene active layer. **(a)** Sensors attached on a glove to record the fingers motion. **(b) **I–V curves by different bending diameter as shown in the inset image. **(c)** Bending and relaxing cycles of the sensor. **(d)** The response and recovery time of the sensor. **(e)** Sensitivity and maximum stretching % along graphene and iron oxide composite blending ration.

Here, ${S}_{R}$ defines the percentage change in the resistance of the device, $\Delta R$ describes the change in resistance, and ${R}_{o}$ is the resistance at normal position. The blending ratio of 1:0 has the highest sensitivity as it has just graphene flakes, but the stretching parameter is low because there are no magnetic iron oxide nano-particles to make them bind together, hence a change in the resistance is substantial and unstable. As shown in Fig. 3, we fabricated the sensor with just graphene as the active layer. It can also work as a stain sensor with a stable behavior, but the stretching behavior is less pronounced and also having the high error in resistance. Figure 3a shows the realization of the device with graphene as the active layer as the strain sensor. The sensors are attached on the glove to record the motion of the fingers. Figure 3b shows the I-V curves of the sensor via different bending diameters as the inset image in this figure. Through finger detection in Fig. 3a, the bending and relaxing cycles are measured as shown in Fig. 3c. Here, its response of 0.3 s and recovery time of 0.45 s are recorded as shown in Fig. 3d. These results show that just graphene flakes can be used as strain sensor, but the stretching percent (%) is less. To increase the stretching parameter and reduce the error, we blend the magnetic iron oxide in graphene with different ratios. When the ratio of the magnetic iron oxide is increased, the stretching performance is increased and the resistance error is reduced. As shown in Fig. 3e, 1:1 was the optimum ratio for the proposed strain sensor with a valuable sensitivity error and high stretching %. Here, the sensor with 1:0 blending ratio has the highest sensitivity error as the reason of the graphene flakes are only used. When we add the magnetic iron oxide, the sensitivity error decreased because it provides connection between flake and flake, and the stretchability also was enhanced. From this analysis, we have selected the graphene and magnetic iron oxide composite blending ratio of 1:1 for the proposed strain sensor.

**Figure 4:**
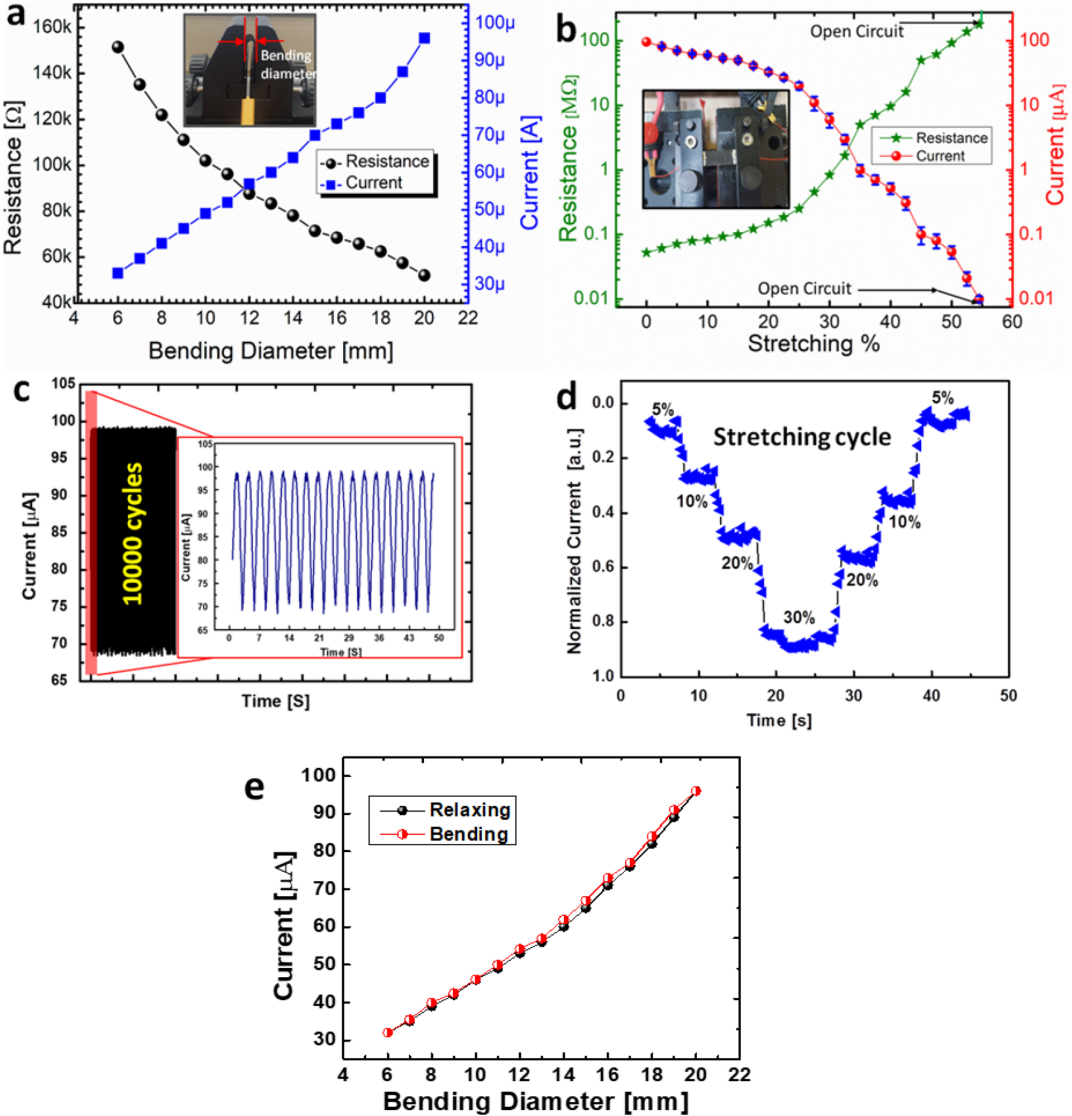
Characterization of the self-healing strain sensor with graphene and magnetic iron oxide composite film. **(a)** Current and resistance changes along different bending diameters as the inset image. **(b)** Current and resistance changes along different stretching % as the inset image. **(c)** 10,000 bending/relaxing cycles. **(d)** Normalized current variation along the stretching cycle of the proposed strain sensor. **(e)** Hysteresis curve of bending and relaxing.

To understand the proposed sensor based on graphene and magnetic iron oxide nano-composite material, the bending effect is measured along different bending diameter as the inset image shown in Fig. 4a. As shown in Fig. 4a, the electrical resistance and current are almost linearly increased under bending diameter down to 6 mm, which confirm the proposed strain sensor can be used in the finger bending detection. For this reason, we attached five sensors on a glove to test the application performance as shown in Fig. 3a, and we came to know that it shows stability and uniformity to the bending. Electrical resistance was changed due to the graphene flakes overlapping area-changing and cracking phenomenon in the nano-composite film of the sensor. The data was calculated for 5 sensors at different bending diameters to investigate the error in resistance and its stability. Very low error is observed and the maximum error is ± 10 Ω via 6 mm bending diameter, which is negligible error. Similarly, the current via different diameter was also checked and there was uniform change in current as shown in Fig. 4a. The bendability test suggests that the proposed strain sensor can be used for the human body motion detection. As shown in Fig. 4b, the sensor was stretched up to 54.4% and there was linear change in resistance and current along different stretching %. After 54.5% stretching, it shows open circuit due to the breakdown of the composite film. However, on relaxing the stretching, it recovers its 100% efficiency due to self-healing. For a stability for a long-term strain endurance cycles, the proposed strain sensor is tested for more than 10,000 bending/relaxing cycles as shown in Fig. 4c and the sensor shows stable behavior without losing its performance. Figure 4d shows the stretching cycle of the proposed strain sensor. Starting from 5% stretching, the sensor was stretched step by step following 10%, 20%, and 30% and then again relaxed with the stretching following 20%, 10%, and 5%. The sensor shows a stable response and recovered to its original position. Figure 4e shows the hysteresis curve of bending and relaxing as it shows stability. For these results, we can say that the proposed sensor can be used as a strain sensor for wearable electronics.

**Figure 5:**
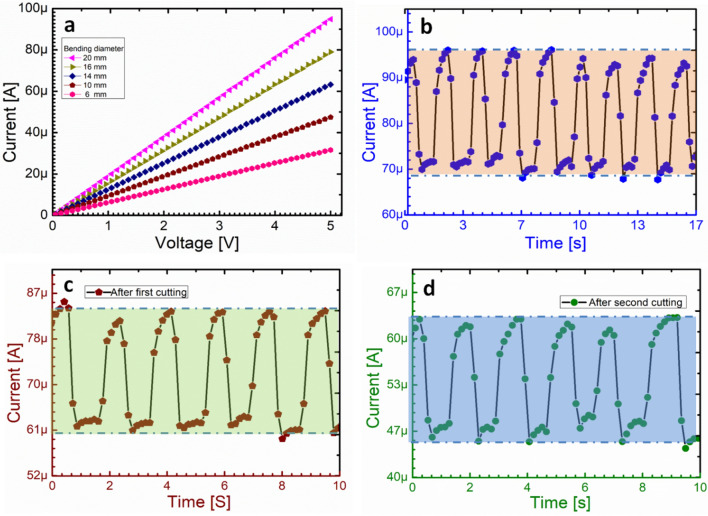
Electrical characterization of the self-healing strain sensor before and after the cutting. **(a)** I–V curves of the proposed self-healing strain sensor at different bending diameter. **(b)** Bending and relaxing cycles before cutting the proposed sensor. **(c)** Bending and relaxing after first cutting and the sensor recovers its 94% efficiency. **(d)** Bending and relaxing after 2nd time cutting and still the proposed sensor is recovered after healing.

The electrical performance of the proposed strain sensor was analyzed on different bending angles using Agilent B1500A Semiconductor Device Analyzer by applying sweeping voltage from 0 to 5 V. Figure 5a shows I–V characteristics over different bending diameter (6 mm, 10 mm, 14 mm, 16 mm, and 20 mm), which shows ohmic relationship between the voltage and the resulting electrical current of the graphene/Fe_2_O_3_ nano-composite. Hence, the proposed sensor can be applicable to measure the different bending angle and strength for the strain applications. For applying the strain sensor in a real-life application, we observed the stretching/releasing cycles for a long period of time, and it shows a good stability with a small change of the normalized resistance during stretching/releasing cycles without any failure as shown in Fig. 5b. The proposed strain sensor has a good mechanical durability against the repeated elongation/relaxation cycles such as human motion. Moreover, the stability of the sensor under static loading is just drifted of 5.7 Ω in the electrical resistance, when the proposed strain sensor was bent down to 8 mm for 20 min. Furthermore, the proposed sensor was placed in the 2-axis motion controlled [STM-2-TBDS] bending machine to test the stretching %. To investigate the recovery % of the proposed strain sensor after cutting it, we cut the strain sensor two times at the same place. After 1st cutting, it recovers 95% and shows stable bending and relaxing cycles as shown in Fig. 5c. After second time cutting at the same place, the proposed sensor recovers its 72% of the initial efficiency as shown in Fig. 5d, but still it can work as a strain sensor with stable bending and relaxing cycles. After 2nd time cutting, the stretching parameter is reduced to 32.5%, which is still a high stretching % as compared to other graphene based strain sensors^51^. From these results. We can say that the proposed strain sensor can be cut and reuse again, and can be used as a self-healing strain sensor. The gauge factor of the device was 271.4 at 35%, hence the proposed sensor is good sensitivity.

**Figure 6:**
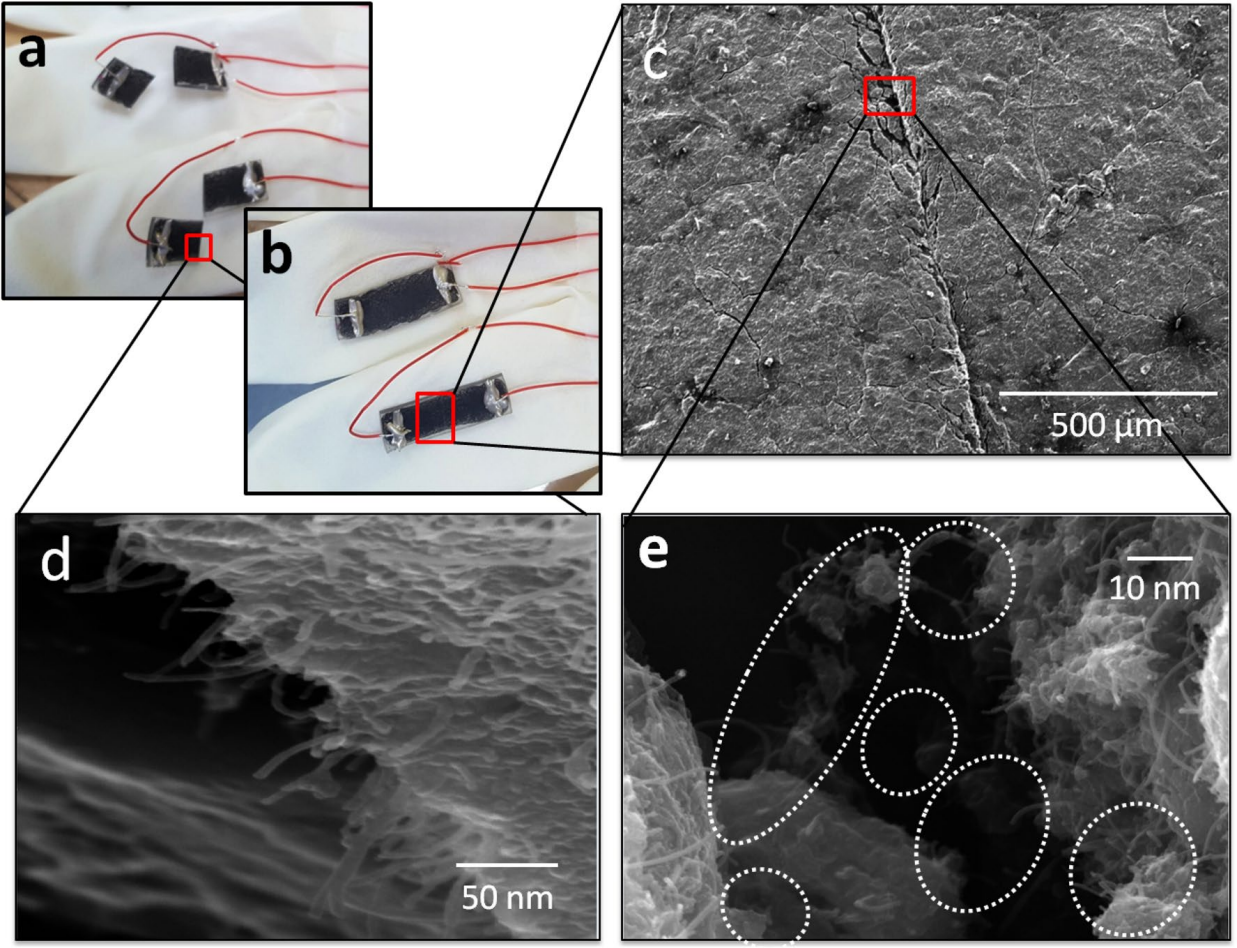
Cutting and healing of the proposed strain sensor. **(a)** Images of the cutting sensors. **(b)** Images of the sensors after self-healing and we can see that the sensors are fully healed (it can see in supplementary movie). **(c)** SEM image of the sensor after self-healing. **(d)** Cross section SEM images of the sensor after cutting it. Here, we can see both the graphene flakes as well magnetic iron oxide. **(e)** SEM image analysis of self-healing of the strain sensor. Here, the thread like structure and magnetic property of the magnetic iron oxide is the main reason of self-healing as shown in the SEM image.

**Figure 7:**
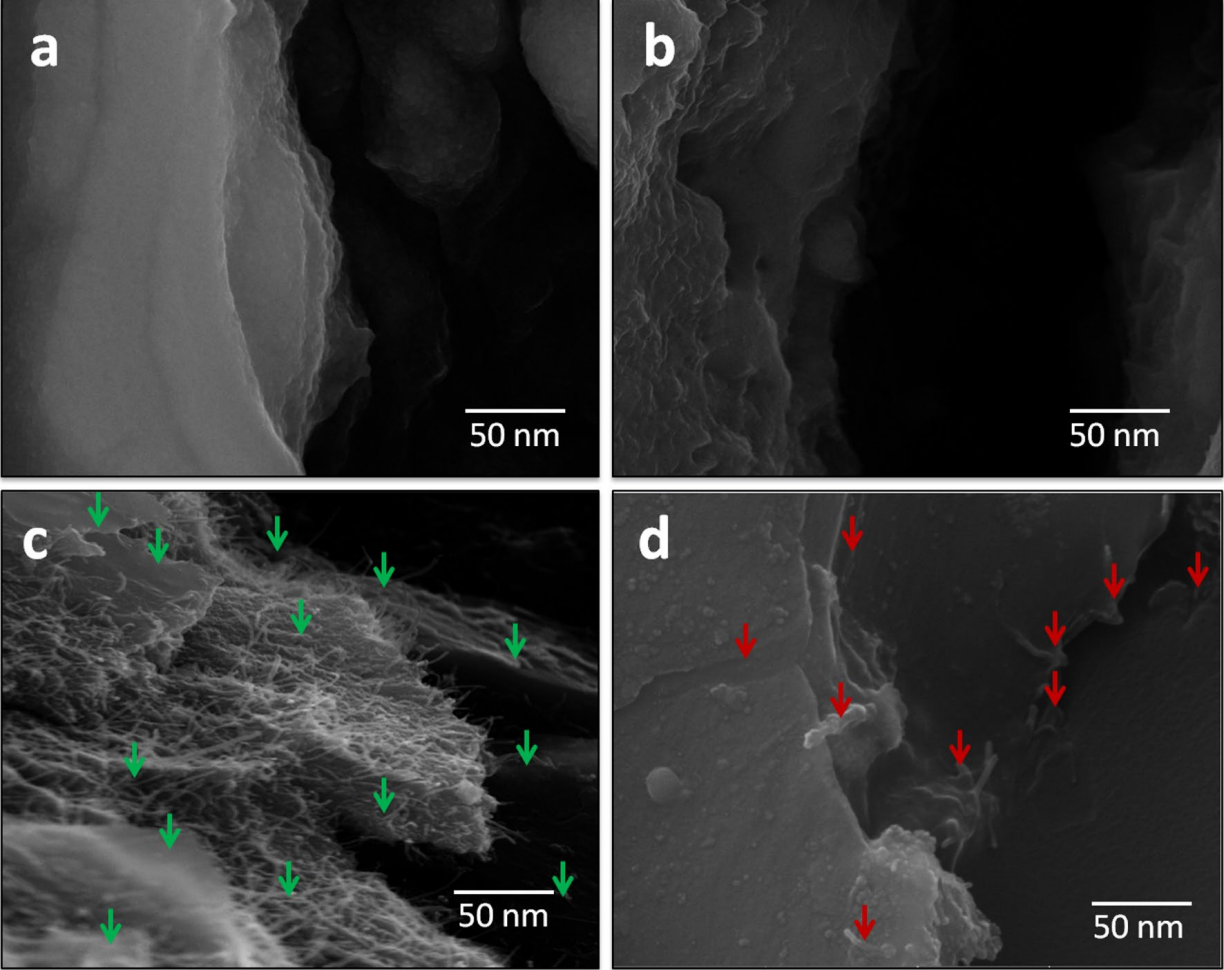
Comparison of the SEM of the films without and with Fe_2_O_3_. **(a)** SEM of the film without Fe_2_O_3_, where, in this SEM image, we can see the graphene flakes, clearly. **(b)** SEM image after cutting the film. Here, we can see the layered structure based on graphene flakes without Fe_2_O_3_. **(c)** SEM image of the composite film can clearly see graphene and Fe_2_O_3_. Here, in this image, the green arrows present the graphene flakes covered with Fe_2_O_3_. **(d)** SEM image of the composite film can also clearly seen graphene and Fe_2_O_3_. Here, we can see the graphene flakes and the red arrows shows the Fe_2_O_3_.

In order to verify the self-healing characteristics of the proposed sensors, we cut the strain sensor in two pieces as shown in Fig. 6a. After combing the two pieces, it heals again without an external treatment (see supplementary movie) due to two reasons; magnetic feature and healing property by the graphene and magnetic iron oxide nano-composite in active layer and by the polyurethane in substrate, respectively, as shown in Fig. 6b. That is why, we are using polyurethane as a substrate which has the self-healing property, and the active layer is recovered by itself due to the presence of the magnetic iron oxide nano-particles. As we have the evidence form SEM images in Fig. 6c-e, the magnetic iron oxide has the thread like structure due to its magnetic property. This results in the self-healing property of the active layer. We can also confirm this behavior from the SEM images of just graphene and with magnetic iron oxide structures. The healing time is depending on the angle at which the sensor parts are placed head to head after 100% cutting as shown in Fig. 6a,b. On varying the healing angle, the response time of healing will be variable. But if the healing angle is constant, the response of healing time will also be constant. Figure 7a,b show the SEM images of just graphene as the active layer. Here, we can see that there are just graphene flakes and no threads. But for the SEM images of the active layer with the graphene and magnetic iron oxide as shown in Fig. 7c,d. We can clearly see the graphene flakes and the magnetic iron oxide thread like structure, which is the main reason for self-healing property of the fabricated strain sensor.

To figure out the sensing mechanism of the strain sensor based on graphene/Fe_2_O_3_ nano-composite active film, the structural changes in the film under different levels of strain were examined to examine the effect of stretchability**.** As shown in Fig. 2a-c, the cracking phenomenon is illustrated on initial stretching, the fractures and the overlapping graphene flakes area variations are firstly occurred on the film. After certain value of strain, the cracking propagation becomes a second dominant process on the surface of active film, due to the crack density and their width are increased as shown in Fig. 2g-l, which corresponds the main reason of the exponential increase in its electrical resistance. On smooth polyurethane case, a large number of the cracks were occurred on the surface of active film. In result, the strain endurance was reduced. Over 15% stretching case, the film was fully broken which indicated open circuit behaviors. In order to overcome the film breaking phenomena on smooth surface over 15% stretching, we have created roughness on the surface of polyurethane. As compare to smooth surface, rough polyurethane surface shows increase in stretchability range from 15 to 54.5%. The small number of cracks were appeared on the active layer during stretchability, which helps to maintain the overlapping area and contact resistance of the graphene flaks network determine the conductivity between the neighboring flakes. During compression or tension strain the overlap area between neighboring flakes becomes smaller or larger, which results in change in the resistance of the film. The magnetic property of Fe_2_O_3_ is utilized in with graphene for the self-healing of the active film. The Fe_2_O_3_ helps to connects the graphene flakes due to its magnetic force property and the stretchability is increased dramatically. Besides that, the polyurethane substrate has also the self-healing property, so due to Fe_2_O_3_ and polyurethane substrate the strain sensor has the self-healing property. The proposed strain sensor can be cut and reuse again and again. When the strain force is applied, the connected joints among the graphene flakes are decreased and the conductive paths are reduced as shown in Fig. 2, and change in electrical resistance is occurred. However, on releasing the strain force, the active film gets recover again due to magnetic iron oxide and polyurethane substrate. These mechanisms suggest that the graphene flakes and magnetic iron oxide composite are suitable for self-healing strain sensor applications.

## Conclusions

In this paper, a novel self-healing high stretchable strain sensor was proposed, which could be operated after cutting it. The proposed strain sensor was fabricated depositing graphene and magnetic iron oxide nano-composite on the engineered self-healable polyurethane substrate through the commercialized inkjet printer. To improve a self-healing function of the active sensing layer, the graphene/magnetic iron oxide composite was applied, the best graphene and magnetic iron oxide composite blending ratio is 1:1. The proposed sensor had a high mechanical property, a good sensitivity towards strain, and an excellent self-healing property due to both the composite material and the polyurethane substrate. The proposed sensors have maintained its operating sensitivity over bending cycles on human fingers and a 94% of its operating sensitivity was recovered even after cutting the sensor. The proposed strain sensor was stretchable up to 54.5%. After cutting the sensor, its stretching factor decrease down to 32.5%. From these results, the proposed sensor could create a novel self-healing wearable electronic devices.

## Supplementary information

Supplementary Information.

Supplementary Video 1.
