# Supplementary material for: Inkjet printed self-healable strain sensor based on graphene and magnetic iron oxide nano-composite on engineered polyurethane substrate
Source: Sci Rep. 2020 Oct 26;10:18234. doi: 10.1038/s41598-020-75175-6 (PMC7589529; doi:10.1038/s41598-020-75175-6)
Supplement: Supplementary file 1 — Supplementary Information. [file 41598_2020_75175_MOESM1_ESM.docx]

**Supplementary Information: Inkjet printed self-healable strain sensor based on graphene and magnetic iron oxide nano-composite on engineered polyurethane substrate**

Gul Hassan^1,2^, Muhammad Umair Khan^1^, Jinho Bae^1*^ and Ahmed Shuja^2^

^1^Department of Ocean System Engineering, Jeju National University, 102 Jejudaehakro, Jeju 63243, South Korea

^2^Centre for Advanced Electronics & Photovoltaic Engineering, International Islamic University, H-10, Islamabad, Pakistan

^*^E-mail: [baejh@jejunu.ac.kr](mailto:baejh@jejunu.ac.kr)


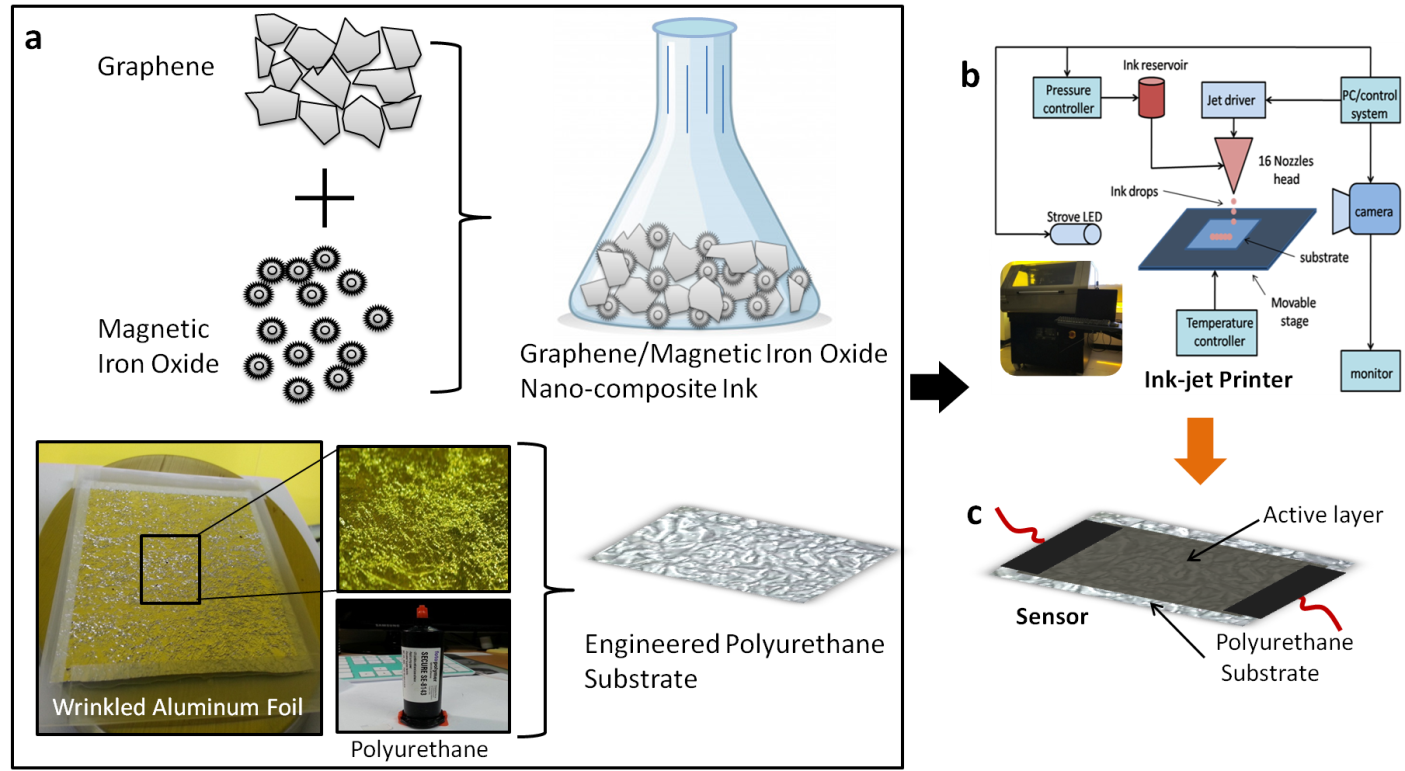


**Figure S1**. Materials preparation and step by step fabrication process. (a) Preparation of the graphene and magnetic iron oxide nano-composite and polyurethane substrate. (b) Inkjet materials printer DMP-3000 and its schematic diagram. (c) The proposed strain sensor.


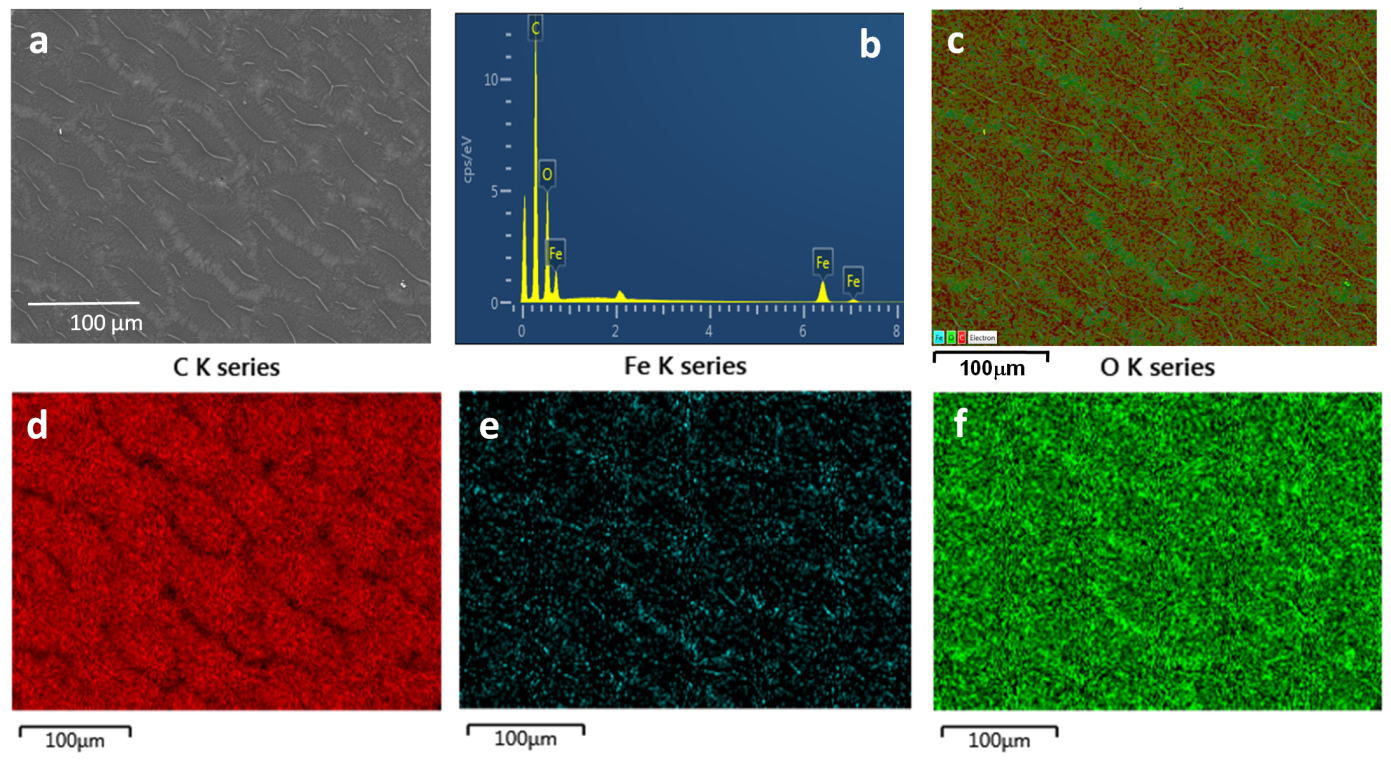


**Figure S2**. (a) EDS mapping SEM image. (b) Map sum spectrum of active layer, that shows that Fe, C and oxygen are present in th self-healing composite active film. (c) Elemental EDS mapping, that shows the presence of both materials in composite film. (d) ESD mapping showing C. (e) ESD mapping showing Fe. (f) ESD mapping showing O.
